# Positive and Negative Attitudes Toward Artificial Intelligence and E-Healthy Diet Literacy Among Adults: A Partial Least Squares Structural Equation Modeling Study

**DOI:** 10.3390/nu18172931

**Published:** 2026-09-07

**Authors:** Çiler Özenir, Canan Altınsoy, Mihrican Çubuk, Fatma Nişancı Kılınç, Biriz Çakır

**Affiliations:** 1 Department of Nutrition and Dietetics, Kırıkkale University, 71100 Kırıkkale, Türkiye; cileraslanalp@kku.edu.tr (Ç.Ö.); fatmanisanci@kku.edu.tr (F.N.K.); birizcakir@kku.edu.tr (B.Ç.); 2 Department of Nutrition and Dietetics, Recep Tayyip Erdoğan University, 53100 Rize, Türkiye; 3 Department of Nutrition and Dietetics, Erzincan Binali Yıldırım University, 24100 Erzincan, Türkiye; mihrican.kacar@erzincan.edu.tr

**Keywords:** artificial intelligence, digital nutrition literacy, e-healthy diet literacy, general attitudes toward artificial intelligence, nutrition literacy, PLS-SEM

## Abstract

**Background/Objectives**: This study aimed to examine the association between e-healthy diet literacy (e-HDL) and general attitudes toward artificial intelligence (AI) among adults. **Methods**: This cross-sectional study included 610 adults aged 19–64 years. Data were collected through face-to-face interviews using a questionnaire consisting of five sections, including sociodemographic characteristics, digital nutrition information practices, AI use in nutrition and dietetics, the e-Healthy Diet Literacy Questionnaire (e-HDLQ), and the General Attitudes toward Artificial Intelligence Scale (GAAIS). Descriptive statistics, validity and reliability analyses, correlation analyses, and Partial Least Squares Structural Equation Modeling (PLS-SEM) were performed. Statistical significance was accepted at *p* < 0.05. **Results**: The mean age of the participants was 33.07 ± 11.36 years, and 50.8% were female. The measurement model demonstrated acceptable reliability and convergent validity. Structural model analysis showed that positive attitudes toward AI were positively associated with e-HDL (β = 0.243, *p* < 0.001), whereas negative attitudes toward AI were negatively associated with e-HDL (β = −0.232, *p* < 0.001). Among the e-HDLQ dimensions, the strongest loading was observed for finding e-healthy diet information (β = 0.844), followed by applying e-healthy diet information (β = 0.641), digital healthy eating literacy (β = 0.636), judging e-healthy diet information (β = 0.308), and understanding e-healthy diet information (β = 0.277). Together, positive and negative attitudes toward AI explained 12.2% of the variance in e-HDL (R^2^ = 0.122), indicating a modest explanatory contribution. **Conclusions**: Positive and negative attitudes toward AI were independently associated with e-HDL among adults. Although the explained variance was modest, AI attitudes may represent one of several factors associated with digital nutrition literacy, warranting further investigation in future studies.

## 1. Introduction

Maintaining an adequate and balanced diet depends not only on individuals having the right nutritional knowledge, but also on their ability to access that knowledge, understand and evaluate it, and apply it to their daily lives. While it has been shown that the level of nutritional knowledge is associated with healthier food choices and positive eating patterns, it is reported that this association is generally weak [1,2]. Nutrition literacy, on the other hand, is a broader concept than simply the level of knowledge; it refers to the set of knowledge and skills necessary for individuals to access, understand, and evaluate nutritional information and translate it into appropriate dietary decisions [3]. As health and nutrition information has increasingly moved online, the core competencies of accessing, understanding, appraising, and applying information described in health-literacy frameworks have also become relevant in digital environments [4]. E-healthy diet literacy (e-HDL) refers to participants’ ability to find, understand, appraise, and apply healthy-eating information obtained from electronic sources in their daily lives [5]. These competencies are particularly important because online nutrition information varies substantially in quality and accuracy, placing users at risk of being misinformed and increasing the need to appraise the source, supporting evidence, and reliability of the information encountered online [6].

Artificial intelligence (AI) is increasingly used in information processing, decision support, monitoring, and personalised service delivery, including within health care. In health-related settings, AI applications have been developed for tasks such as diagnostic support, patient monitoring, clinical decision support, and treatment planning [7,8,9]. Perceived usefulness and expected health benefits may be important factors associated with individuals’ willingness to engage with AI-enabled health technologies [8]. More recently, research has begun to explore generative AI and large language models as components of personalised nutrition-oriented recommendation systems [10]. At the same time, the increasing use of AI has raised concerns regarding misinformation, algorithmic bias, accountability, privacy, and the potential consequences of erroneous outputs in high-stakes settings such as health care. These concerns have also contributed to broader ethical and regulatory debates surrounding the development and use of AI systems [11].

From a theoretical perspective, the relationship between attitudes toward artificial intelligence (AI) and e-HDL can be explained through technology acceptance and health information processing perspectives. The Technology Acceptance Model (TAM) proposes that individuals’ perceptions of a technology, particularly perceived usefulness and perceived ease of use, influence their intention to accept and use the technology [12]. Similarly, the Unified Theory of Acceptance and Use of Technology (UTAUT) identifies performance expectancy, effort expectancy, social influence, and facilitating conditions as important determinants of technology acceptance and use [13]. Although these models were not developed specifically to explain e-HDL, they provide a theoretical basis for proposing that individuals’ evaluations of AI may influence their tendency to engage with and use digital platforms. In this context, more favourable evaluations of AI may facilitate engagement with AI-supported health and nutrition information, thereby increasing individuals’ willingness to actively seek, understand, evaluate, and apply such information, which are core competencies underlying e-HDL. Conversely, negative evaluations of AI may reduce individuals’ willingness to engage with AI-supported information sources or lead them to approach such information more cautiously. Accordingly, attitudes toward AI may be associated with individuals’ engagement with and evaluation of AI-mediated health and nutrition information. While positive attitudes may facilitate engagement with AI-supported information sources, negative attitudes may reflect a more cautious approach. Therefore, positive and negative AI attitudes may theoretically be associated with e-HDL in different directions or to different degrees.

General attitudes toward AI may shape how individuals approach health and nutrition information generated or supported by AI. Perceiving AI as useful and beneficial may encourage engagement with AI-supported information sources, whereas concerns regarding reliability, privacy, ethical implications, or potential harm may be associated with more cautious or avoidant engagement. Importantly, positive and negative attitudes toward AI are distinct subscales rather than opposite ends of a single continuum. Individuals may therefore recognise the potential personal or societal benefits of AI while simultaneously expressing concerns about its reliability, ethical implications, privacy, and broader social consequences [14,15]. These attitudes may also be associated with the competencies required to navigate digital nutrition information. More positive attitudes toward AI may encourage individuals to engage with emerging digital health and nutrition tools, potentially increasing their exposure to and use of electronic nutrition information. Conversely, stronger digital appraisal competencies may also make individuals more attentive to the limitations, inaccuracies, and risks of AI-generated content, suggesting that greater e-HDL need not correspond solely to uniformly positive attitudes toward AI. Consistent with this possibility, two recent cross-sectional studies reported that higher levels of digital or e-HDL were associated with more favourable general attitudes toward AI [16,17]. However, both studies used the brief, unidimensional Artificial Intelligence Attitude Scale-Short Form (AIAS-4), which provides a single overall measure of general attitudes toward and acceptance of AI. As a result, it does not differentiate between positive evaluations of AI and concerns about its potential risks. Consequently, potentially different associations of e-HDL with positive and negative AI attitudes could not be examined separately in these studies [16,17]. Accordingly, this study aimed to investigate the associations between positive and negative attitudes toward AI and e-HDL among adults. By modelling positive and negative AI attitudes as distinct constructs, it assessed whether these two subscales showed different relationships with e-HDL. The structural model additionally examined potential indirect and conditional pathways that might help explain or modify these associations. Given the limited evidence in this field, the study aimed to provide a more nuanced examination of the factors associated with e-HDL in the context of AI.

## 2. Materials and Methods

### 2.1. Participants and Procedures

This cross-sectional study was conducted among 610 adults aged 19–64 years. The study protocol was approved by the Erzincan Binali Yıldırım University Human Research Ethics Committee for Health and Sports Sciences (Decision No. 10/7, 24 October 2025). Written informed consent was obtained from all participants who voluntarily agreed to participate in the study, in accordance with the principles of the Declaration of Helsinki [18].

The required sample size was calculated using G*Power software (version 3.1.9.7). Assuming a statistical power of 85% and a two-sided significance level of 0.05, the minimum required sample size was estimated to be 445 participants. Participants were recruited using a convenience sampling method. The study was conducted in Kırıkkale, Türkiye, between November and December 2025. Participants were excluded if they had physical or cognitive disabilities that could interfere with questionnaire completion, were illiterate, were pregnant or breastfeeding, or were dietitians or undergraduate students in the Department of Nutrition and Dietetics.

The researchers developed questionnaire consisted of five sections and was administered through face-to-face interviews. The first section collected general participant characteristics, including date of birth, sex, marital status, educational level, income status, presence of chronic diseases, and anthropometric characteristics. Body weight and height were self-reported, and body mass index (BMI) was calculated by the researchers. BMI is classified according to World Health Organization (WHO) criteria as underweight: <18.5 kg/m^2^, normal weight: 18.5–24.9 kg/m^2^, overweight: 25.0–29.9 kg/m^2^, and obesity: ≥30.0 kg/m^2^ [19]. The second and third sections assessed participants’ healthy eating information and practices on digital platforms, as well as their opinions regarding the use of AI in the field of Nutrition and Dietetics. In the fourth section, participants completed the e-Healthy Diet Literacy Questionnaire (e-HDLQ), while the fifth section included the General Attitudes toward Artificial Intelligence Scale (GAAIS).

#### 2.1.1. e-Healthy Diet Literacy Questionnaire (e-HDLQ)

The e-Healthy Diet Literacy Questionnaire (e-HDLQ) was originally developed by Duong et al. and first published in 2020 [5]. Its Turkish validity and reliability were established by Onbaşı and Türker in 2023 [20]. The Turkish version consists of 15 items grouped into five dimensions: Finding e-Healthy Diet Information, Understanding e-Healthy Diet Information, Judging e-Healthy Diet Information, Applying e-Healthy Diet Information, and Digital Healthy Diet Literacy. The total score is calculated by summing the scores obtained from all items. The maximum possible score is 71, with higher scores indicating higher levels of e-HDL. In the original development study, the e-HDLQ demonstrated a Cronbach’s alpha coefficient of 0.64. In the Turkish validation study, the overall Cronbach’s alpha coefficient was 0.77 [20].

#### 2.1.2. General Attitudes Toward Artificial Intelligence Scale (GAAIS)

The General Attitudes toward Artificial Intelligence Scale (GAAIS) was developed by Schepman and Rodway (2020) to assess participants’ general attitudes toward AI [14]. The Turkish validity and reliability study of the scale was conducted by Kaya et al. (2022) [21]. The scale consists of 20 items and two subscales: the Positive GAAIS and the Negative GAAIS. The Positive GAAIS comprises 12 items, whereas the Negative GAAIS comprises 8 items. All items are rated on a five-point Likert-type scale ranging from 1 (strongly disagree) to 5 (strongly agree). The Negative GAAIS items are reverse-scored, and separate mean scores are calculated for the positive and negative subscales. Thus, the mean score for each subscale ranges from 1 to 5, with higher scores indicating a more positive attitude toward AI. In the original validation study, Cronbach’s alpha coefficients were 0.88 for the Positive GAAIS and 0.83 for the Negative GAAIS. In the Turkish validation study, Cronbach’s alpha coefficients were 0.82 for the Positive GAAIS and 0.84 for the Negative GAAIS, while the corresponding split-half reliability coefficients were 0.77 and 0.83, respectively [21]. In the present measurement model, three items from the Negative GAAIS subscale (items 13, 14, and 16) were removed because of low factor loadings, resulting in a final 17-item model comprising 12 Positive GAAIS items and 5 Negative GAAIS items.

### 2.2. Statistical Analysis

The data obtained in the study were analyzed using SPSS (Statistical Package for Social Sciences) version 25.0, Databeeg version 1.0, and SmartPLS version 4 programs. Descriptive statistics such as mean ($\bar{X}$), standard deviation (SD), frequency, and percentage were used to evaluate the data. Normality analysis was performed to examine the distribution characteristics of the dataset. Conformity to normal distribution was assessed by examining Q-Q Plot graphs, one of the graphical methods. Normality was assessed visually using Q-Q Plot graphs [22], and skewness and kurtosis coefficients were also calculated. Following the literature, values within the ±3 range were considered indicative of approximate normality [23]. Normality assessment was conducted to verify the assumption for Pearson correlation analysis. Given that the normality assumption was satisfied, Pearson correlation analysis was employed to examine the relationships among the variables. To test the proposed research model, Partial Least Squares Structural Equation Modeling (PLS-SEM) was adopted due to its suitability for explaining and predicting target constructs in a relatively complex structural model. SmartPLS software was used to test the research model, assess the validity and reliability of the measurement model, and examine the structural relationships. Furthermore, PROCESS Macro was utilized to evaluate the mediating and moderating effects proposed in the research hypotheses. The mediation and moderation analyses were conducted based on theoretically derived hypotheses concerning the potential roles of participant characteristics and AI-related factors in the relationships among the study variables. PLS-SEM analyses were performed in two stages. In the first stage, the measurement model was assessed. Its reliability was evaluated using Cronbach’s alpha (α) and composite reliability (CR) coefficients. Convergent validity was assessed through average variance extracted (AVE) values, while discriminant validity was tested using the Fornell–Larcker criterion and the heterotrait–monotrait ratio (HTMT). Following the recommended thresholds, Cronbach’s alpha and rho_A values were set above 0.60 [24], CR values above 0.70 [25], and AVE values at or above 0.40 [24]. The HTMT represents the ratio of the average inter-item correlations across different constructs to the geometric mean of the average inter-item correlations within the same construct [26], and was required to be 0.90 or lower [24]. In the second stage, the structural model was evaluated by examining path coefficients, the coefficient of determination (R^2^), effect sizes (f^2^), and predictive relevance (Q^2^). R^2^ measures the variance explained in each endogenous construct, thus indicating the model’s explanatory power (also referred to as in-sample predictive power), with values ranging from 0 to 1—higher values denoting greater explanatory power [24]. The f^2^ value indicates the strength of the relationship between each independent variable and the dependent variable, with values of 0.02, 0.15, and 0.35 representing small, medium, and large effect sizes, respectively [27]. Predictive relevance was assessed using Stone–Geisser’s Q^2^ coefficient, where a value greater than zero suggests that the model has predictive relevance for the relevant endogenous construct. Moreover, Q^2^ values exceeding 0, 0.25, and 0.50 indicate small, medium, and large predictive accuracy of the PLS path model [24]. The significance of the relationships was tested using the bootstrapping procedure. In addition to the hypothesized structural paths, the effects of BMI, number of diagnosed diseases, trust in the accuracy of AI-generated healthy nutrition information, and acceptance of an AI-generated nutrition/diet plan on e-HDL were also examined. Consequently, only statistically significant paths are reported in the final structural model. The threshold for statistical significance was set at *p* < 0.05.

## 3. Results

A total of 610 adults aged 19–64 years participated in the study, with a mean age of 33.1 ± 11.4 years. Of the participants, 50.8% were female, 60.0% were university graduates, 50.7% were married, and 44.3% reported that their income was equal to their expenses. The mean BMI was 25.3 ± 5.3 kg/m^2^; 44.4% of the participants had normal weight, 33.0% were overweight, and 15.7% had obesity. A diagnosed disease was reported by 26.6% of the participants. The mean Positive and Negative GAAIS scores were 39.6 ± 10.2 and 16.2 ± 4.4, respectively. The mean total e-HDL score was 45.0 ± 6.8. The mean scores for the finding, understanding, judging, applying, and digital healthy diet literacy dimensions were 7.1 ± 2.8, 16.5 ± 2.3, 7.2 ± 2.3, 4.1 ± 2.0, and 10.2 ± 2.8, respectively (Table 1).

Regarding digital nutrition information practices, most participants reported assigning at least some importance to the scientific credibility of online information (87.3%), and more than half followed dietitians on digital platforms (55.4%). Digital information was perceived as having at least some influence on healthy lifestyle habits by 77.6% of participants. The most frequently searched topics were the health effects of foods (52.5%), healthy recipes (50.2%), weight-loss-related nutrition information (39.2%), and food supplements (37.9%). Knowledge of AI applications in nutrition and dietetics was generally limited, with 72.0% reporting no or very little knowledge. Although 59.3% trusted the accuracy of AI-generated healthy nutrition information, only 34.4% were willing to follow an AI-generated diet plan. Views regarding the wider use of AI-supported nutrition and dietetics applications were mixed, with 39.2% responding positively and 39.8% remaining undecided (Table 2).

Table 3 presents the Pearson correlation coefficients between the study variables. Age was positively correlated with understanding e-healthy diet information (r = 0.118, *p* = 0.004) and BMI (r = 0.348, *p* < 0.001), and negatively correlated with the Positive and Negative GAAIS scores (r = −0.135, *p* = 0.001 and r = −0.119, *p* = 0.003, respectively). The total e-HDL score was positively correlated with the Positive GAAIS score (r = 0.222, *p* < 0.001) and negatively correlated with the Negative GAAIS score (r = −0.165, *p* < 0.001). Among the e-HDL dimensions, judging e-healthy diet information showed the strongest correlation with the Positive GAAIS score (r = 0.303, *p* < 0.001). The Positive and Negative GAAIS scores were not significantly correlated with each other (r = −0.018, *p* = 0.661).

Table 4 presents the reliability and convergent validity results of the measurement model. Cronbach’s alpha (α) coefficients ranged from 0.670 to 0.914, rho_A values from 0.701 to 0.915, CR values from 0.771 to 0.917, and AVE values from 0.418 to 0.811. All Cronbach’s alpha and rho_A values exceeded the recommended threshold of 0.60, CR values exceeded 0.70, and AVE values exceeded 0.40.

The factor loadings of the items ranged from 0.423 to 0.913, all above the acceptable threshold of 0.40, indicating adequate convergent validity. Three GAAIS items with low factor loadings (items 13, 14, and 16) were removed from the scale, with factor loadings of [0.147], [0.084], and [0.230], respectively. Following item removal, the final GAAIS comprised 17 items, including 12 items in the Positive GAAIS subscale and 5 items in the Negative GAAIS subscale. The remaining five items continued to represent the negative-attitude construct, although the reduced item composition should be considered when interpreting this subscale.

Table 5 presents the discriminant validity results based on the HTMT ratios and the Fornell–Larcker criterion. The HTMT ratios ranged from 0.096 to 0.610 and were below the recommended threshold of 0.90. In addition, the square root of the AVE for each construct exceeded its correlations with the other constructs, supporting discriminant validity. The variance inflation factor (VIF) values for all indicators ranged from 1.263 to 2.853, remaining below the threshold of 5 and indicating no problematic collinearity. The VIF values are provided in the Supplementary Materials.

Table 6 presents the coefficient of determination (R^2^), effect size (f^2^), and predictive relevance (Q^2^) values for the structural model. The R^2^ value of the research model was 0.122, indicating that the independent variables explained approximately 12.2% of the variance in e-HDL. The adjusted R^2^ value was 0.120, which was very close to the R^2^ value. The Q^2^ value was greater than zero (Q^2^ = 0.109), indicating that the model had predictive relevance. The f^2^ values (0.061 and 0.067) indicated that the effect sizes of the independent variables on e-Healthy Diet Literacy were small.

**Figure 1:**
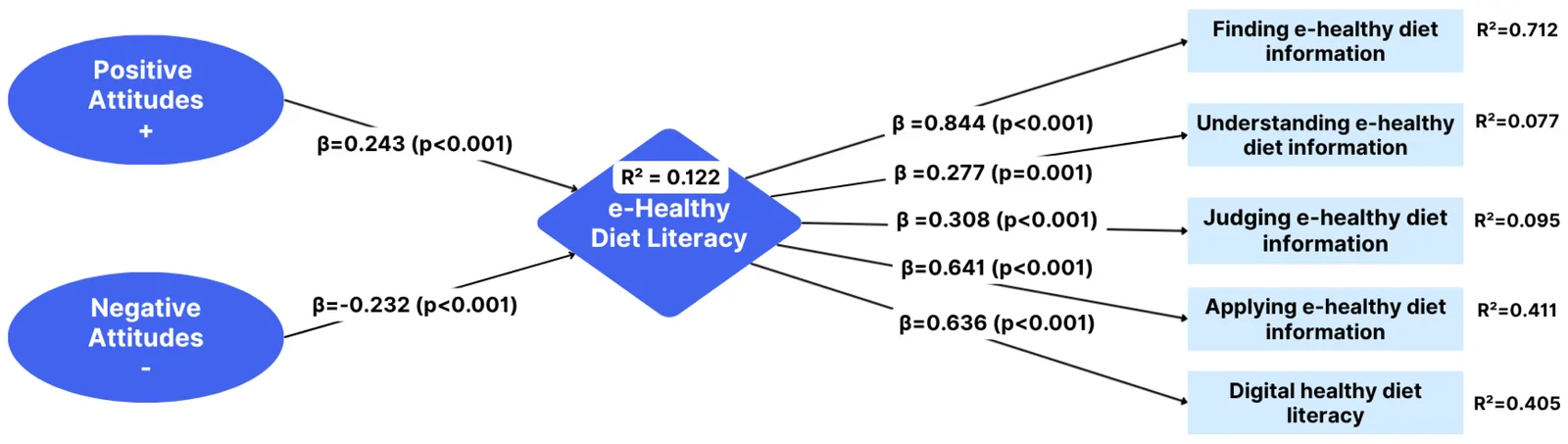
Structural model of the associations between AI attitudes and e-HDL.

Figure 1 presents the research model and the standardized path coefficients from the structural model analysis. Positive and negative GAAIS attitudes were modeled as predictors of e-HDL, and the five e-HDLQ dimensions were examined within the structural model. The coefficients shown on the paths represent standardized β values, and R^2^ values indicate the proportion of variance explained in the corresponding endogenous constructs. Positive attitudes toward AI showed a significant positive association with e-HDL (β = 0.243, *p* < 0.001), whereas negative attitudes showed a significant inverse association (β = −0.232, *p* < 0.001). When the dimensions of e-HDL were examined, the highest path coefficient was observed for finding e-healthy diet information (β = 0.844; R^2^ = 0.712), followed by applying e-healthy diet information (β = 0.641; R^2^ = 0.411), digital healthy diet literacy (β = 0.636; R^2^ = 0.405), judging e-healthy diet information (β = 0.308; R^2^ = 0.095), and understanding e-healthy diet information (β = 0.277; R^2^ = 0.077).

According to the structural model results, negative attitude had a statistically significant and negative effect on e-HDL (β = −0.232, t = 6.229, *p* < 0.001). Accordingly, an increase in negative attitude was associated with a decrease in e-HDLQ. Positive attitude was found to have a statistically significant and positive effect on e-HDL (β = 0.243, t = 7.692, *p* < 0.001). This finding indicates that an increase in positive attitude was associated with an increase in e-HDL. Overall, the findings revealed that both negative and positive attitude variables significantly predicted e-HDL (Table 7).

The mediation analyses conducted using PROCESS Macro Model 4 revealed that BMI and number of diagnosed diseases did not have a significant mediating effect on the relationship between positive attitudes towards AI and e-HDL (Table 8).

Two moderation analyses were conducted using PROCESS Macro Model 1. The first model examined whether acceptance of implementing an AI-generated nutrition/diet plan moderated the relationship between positive attitudes toward AI and e-HDL, whereas the second model examined whether trust in the accuracy of AI-generated healthy nutrition information moderated the same relationship. As shown in Table 9, neither acceptance of implementing an AI-generated nutrition/diet plan nor trust in the accuracy of AI-generated healthy nutrition information had a significant moderating effect on the relationship between positive attitudes toward AI and e-HDL.

## 4. Discussion

This study contributes to the emerging literature by examining the associations between positive and negative attitudes toward AI and e-HDL among adults. The principal finding was that both dimensions of AI attitudes were independently associated with e-HDL: more positive attitudes were associated with higher e-HDL, whereas more negative attitudes were associated with lower e-HDL. However, AI attitudes explained only 12.2% of the variance in e-HDL, indicating that they represent a modest component of the broader factors associated with digital nutrition literacy. The corresponding f^2^ values for positive and negative AI attitudes were also small, further indicating that their participant contributions to e-HDL were limited despite statistical significance. Given the cross-sectional design, the direction of these associations remains uncertain and causal interpretations are not warranted [28,29]. Similarly, Adiyan and Otay Lule reported a moderate positive association between total e-HDL and overall AI attitudes among university students, which remained significant after adjustment for age, sex, BMI, and body appreciation [17]. However, their study used a unidimensional AI-attitude measure and considered only total e-HDL, whereas the present study distinguished positive and negative AI attitudes within the same structural model [17]. By distinguishing positive and negative AI attitudes, the present study provides a more differentiated assessment of their relationships with e-HDL. Delibasi et al. likewise reported a modest positive bivariate association between eHealth literacy and overall AI attitudes among nursing students, although eHealth literacy did not independently predict AI attitudes in their adjusted model. Although conceptually related, eHealth literacy and e-HDL are not equivalent constructs, which limits direct comparison [30]. Direct quantitative comparison is limited by differences in populations, literacy measures, AI-attitude instruments, and analytical approaches across studies. Based on the current findings, it can be interpreted that participants with more positive attitudes toward AI may be more willing to engage with digital technologies when seeking nutrition information. Greater openness to AI may encourage the exploration and use of AI-supported nutrition resources, thereby increasing opportunities to access, understand, appraise, and apply digital nutrition information [28,29]. One possible interpretation is that the observed association reflects differences in engagement with digital technologies; however, engagement was not directly assessed, and the reverse direction is equally plausible. Participants with stronger e-HDL may also feel more confident evaluating AI-supported nutrition information and consequently report more positive attitudes toward AI [5,17]. Concerns regarding reliability, transparency, privacy, or other implications of AI may also be associated with lower willingness to engage with AI-assisted nutrition tools [31,32]. Technology-acceptance research provides a useful, although indirect, framework for interpreting this association. In an AI-specific application of the Technology Acceptance Model, perceived usefulness was positively associated with attitudes toward AI use, while attitudes toward use were strongly associated with behavioural intention. These findings support the broader relevance of attitudinal processes to engagement with AI technologies. However, e-HDL reflects competencies in accessing, understanding, appraising, and applying digital nutrition information rather than technology acceptance or behavioural intention; therefore, TAM should be regarded as a contextual framework for the present findings rather than as a mechanism directly tested in this study [33]. Longitudinal or experimental studies are needed to clarify the direction and mechanisms of the observed relationships [34]. Unlike previous studies relying mainly on bivariate correlations or conventional regression [16,17,30], the present analysis simultaneously modelled positive and negative AI attitudes, allowing their unique associations with e-HDL to be examined. This approach enabled their unique associations with e-HDL to be examined within the same structural model, providing a more differentiated understanding of how positive and negative attitudes toward AI are independently related to e-HDL. The present findings are broadly consistent with recent evidence linking literacy-related competencies to more positive attitudes toward AI.

Among nursing students, Boztepe et al. found that overall AI literacy was moderately associated with positive attitudes toward AI. In multivariable analyses, the usage and judging subscales of AI literacy independently predicted Positive GAAIS scores, with judging emerging as the strongest predictor [35]. Although AI literacy and e-HDL are distinct constructs, these findings provide related evidence that evaluative competencies may be relevant to attitudes toward AI. In the present study, the judging dimension of e-HDL showed the strongest positive correlation with positive AI attitudes, although this pattern should be interpreted descriptively because the correlation coefficients were not formally compared. Participants with stronger e-HDL may therefore perceive themselves as better able to evaluate AI-supported nutrition information, which may be associated with more positive attitudes toward its use [34,36]. However, the cross-sectional design does not establish the direction of this relationship, and greater engagement with AI-supported tools could also be associated with higher e-HDL. Boztepe et al. also reported a more complex pattern for reverse-scored negative attitudes. Although overall AI literacy was not significantly associated with this subscale, AI usage and ethical literacy were associated with fewer negative attitudes, whereas the judging domain was associated with greater recognition of AI-related drawbacks in the multivariable model [35]. This pattern is consistent with the possibility that greater evaluative competence may coexist with both appreciation of AI’s potential benefits and awareness of its limitations, rather than producing uniformly positive perceptions. Accordingly, critical appraisal and positive attitudes toward AI should not be regarded as mutually exclusive [35].

General attitudes toward AI and trust in AI-generated health information are related but distinct constructs [31,37]. Positive attitudes toward AI reflect openness to AI and perceptions of its potential benefits, whereas trust concerns confidence in the accuracy and reliability of specific AI-generated outputs. Consistent with this distinction, trust in the accuracy of AI-generated healthy nutrition information did not significantly moderate the association between positive attitudes toward AI and e-HDL in the present study. Thus, higher positive attitude scores should not be interpreted as indicating unconditional trust in AI-generated nutrition information [31,37,38]. Previous evidence also suggests that trust is more closely related to AI literacy, perceived usefulness, and behavioural intention than to broader health-literacy competencies [37]. Positive attitudes toward AI should therefore not be equated with uncritical reliance, and future studies should examine attitudes, trust, perceived usefulness, behavioural intention, and actual AI use as related but distinct constructs [35,37,39].

Several descriptive findings provided additional context for participants’ engagement with digital nutrition information and AI. Although most participants considered the scientific credibility of information on digital platforms important, only a small proportion rated their knowledge of AI applications in nutrition and dietetics as good or very good. At the sample level, this pattern may indicate that valuing scientifically credible information does not necessarily coincide with greater familiarity with AI applications. Related evidence from digital health research has similarly shown that frequently used information sources are not always those perceived as most trustworthy [38]. This distinction is also relevant to AI literacy, which encompasses not only the use of AI tools but also the knowledge and critical awareness needed to evaluate their outputs and limitations [40]. However, because scientific credibility and AI knowledge were assessed using separate self-reported items, this interpretation remains exploratory. Future educational approaches may therefore consider both digital nutrition-information appraisal and AI-related competencies when addressing the use of AI-generated nutrition information [40,41,42].

Another notable descriptive finding was that more than half of the participants expressed confidence in the accuracy of AI-generated healthy nutrition information, whereas only about one-third were willing to follow an AI-generated diet plan. This pattern may reflect a distinction between perceived trustworthiness and willingness to rely on AI-generated advice, although no individual-level relationship can be inferred because these constructs were assessed using separate self-reported items. This interpretation is consistent with conceptual accounts distinguishing trustworthiness, trust, and trusting behaviour as related but distinct constructs [43]. Evidence from digital healthcare and personalised nutrition further suggests that trust and intention to use may depend on factors such as perceived risk, privacy, data quality, perceived usefulness, previous experience, and professional oversight [37,44,45,46]. The relatively high proportion of undecided responses regarding AI-supported nutrition applications may therefore reflect uncertainty or ongoing appraisal of potential benefits and risks rather than unequivocal rejection, although the underlying reasons were not assessed. These findings support the importance of transparent communication about system performance, limitations, data use, and professional oversight when AI-generated nutrition information is used in practice [37,39,47].

The proportions of participants supporting the wider use of AI-assisted applications in nutrition and dietetics (39.2%) and remaining undecided (39.8%) were nearly identical. This distribution reflects substantial uncertainty alongside support for wider AI use, rather than a clear predominance of either acceptance or rejection. A longitudinal Danish study similarly identified considerable uncertainty toward AI in healthcare and found that, among initially uncertain participants, reported ChatGPT use was associated with subsequent perceptions that the benefits of healthcare AI outweighed its risks [48]. Although this observational association does not establish causality, it indicates that perceptions of AI may change over time and may vary with experience of AI tools. Importantly, undecided responses in the present study should not be equated with ambivalence, neutrality, or implicit acceptance, as the reasons underlying these responses were not assessed.

The practical relevance of evaluative competence is also illustrated by evidence that the quality of AI-generated nutrition information varies across tasks and contexts. Haman et al. (2024) found that ChatGPT provided reasonably consistent estimates for several basic nutritional parameters, although accuracy varied across nutrients and the findings could not be extended to personalised dietary recommendations, particularly for individuals with chronic conditions [49]. Similarly, Liao et al. (2024) reported that ChatGPT-generated dietary advice performed well in readability but was weaker in understandability, practicality, completeness, and fulfilment of nutrition-literacy indicators [50]. These findings indicate that accessible or well-presented AI-generated nutrition information may still require critical appraisal and, where appropriate, professional verification.

Although both positive and negative attitudes toward AI were significantly associated with e-HDL, the structural model explained only a modest proportion of its variance. Notably, the positive and negative GAAIS subscales were not significantly correlated in the present study. This finding is consistent with the original conceptualisation of the GAAIS as comprising two distinct subscales: the positive subscale primarily reflects perceived personal and societal utility, whereas the negative subscale captures concerns and negative emotional responses toward AI [14,15]. The original validation study further showed that participants may simultaneously recognise the benefits of AI while expressing discomfort about applications involving ethical considerations or complex human judgement [14]. Subsequent validation studies on Italian and Hungarian samples have likewise supported the two-factor structure [51,52].

The modest explanatory power of the present model is also consistent with evidence that e-health literacy is shaped by a broad range of behavioural, psychological, sociodemographic, and health-related factors, including internet use, education, income, perceived usefulness, self-efficacy, and health status [53]. AI-specific characteristics not included in the present model, such as previous AI experience, perceived usefulness, digital self-efficacy, innovativeness, or general confidence in using technology, may also account for additional variation [30,35,37]. This interpretation is further supported by technology-acceptance frameworks, which conceptualise engagement with health technologies as a multifactorial process involving perceived usefulness, ease of use, self-efficacy, trust, social influence, and facilitating conditions [54]. Thus, AI attitudes appear to represent only one part of a broader set of factors associated with e-HDL. Future longitudinal and theoretically grounded models should examine these factors alongside general health literacy, digital competence, and socioeconomic resources.

In the theoretical mediation analyses, neither BMI nor the number of diagnosed diseases showed a statistically significant indirect effect on the association between positive attitudes toward AI and e-HDL. Similarly, neither willingness to accept an AI-generated nutrition/diet plan nor trust in the accuracy of AI-generated healthy nutrition information significantly moderated this association. These findings do not support the proposed indirect or conditional pathways in the present sample. Trust and acceptance may reflect more specific responses to AI-generated information or recommendations and may therefore occupy different positions within models of AI-related information use [37,41]. However, the absence of statistically significant indirect or interaction effects should not be interpreted as evidence that these constructs are irrelevant. Limited contextual evidence regarding BMI is available from a cross-sectional study among older adults in Germany. In that study, BMI category and the presence of a chronic disease or long-term health problem were not significantly associated with overall digital health literacy or its information-seeking and information-appraisal subscales after adjustment. Instead, internet use emerged as the most prominent correlate, while self-reported general health were also associated with digital health literacy [55]. However, digital health literacy and e-HDL are distinct constructs, and differences in study populations and analytical approaches limit direct comparison. Evidence regarding the role of diagnosed disease in these relationships also remains limited and methodologically heterogeneous. Longitudinal studies are therefore needed to determine whether BMI, disease status, trust, acceptance, or more proximal behavioural and technology-related factors play indirect or conditional roles in the association between positive AI attitudes and e-HDL.

Having a diagnosed disease and possessing the competencies required to navigate digital health information are therefore related but not equivalent [56]. A recent meta-analysis similarly found that the presence of disease or elevated disease risk was associated with lower eHealth Literacy Scale (eHEALS) scores; however, the included studies were cross-sectional and did not examine disease presence within the mediation pathway tested in the present study [53]. Having a diagnosed disease may increase health-information and self-management demands without necessarily enhancing the competencies required to locate, judge, and apply digital nutrition information [57]. The null indirect effect may also reflect the limited specificity of the disease variable, which distinguished only between participants with and without a diagnosed disease and did not capture disease type, severity, duration, or management requirements. Accordingly, disease presence may be too broad a marker to represent an intermediate pathway between positive AI attitudes and e-HDL. Because this specific mediation pathway has not been examined previously, the available evidence should be regarded as contextual rather than direct support.

Trust in AI-generated nutrition information did not significantly moderate the association between positive attitudes toward AI and e-HDL. This finding should not be interpreted as indicating that trust is irrelevant to AI-supported health-information use. Cross-national evidence suggests that trust in AI-generated health information is more closely related to AI literacy, performance expectancy, and intention to use than to general health-literacy competencies [37]. Similarly, technology-acceptance research has identified trust as a potential mediator of behavioural intention rather than a moderator of broader attitudinal relationships [58]. These findings raise the possibility that trust may occupy a different position within models of AI use than the one tested here, although this interpretation remains tentative. Measurement may provide an additional explanation. Trust in AI is context dependent and may encompass distinguishable evaluations of accuracy, reliability, transparency, privacy, professional competence, role alignment, and usability. Consistent with this multidimensional view, a recently developed instrument for dietitians identified Data Security, Professional Competence, Scientific Accuracy, and Usability as distinct subscales of trust in dialogue-based AI [47]. Although these dimensions cannot be assumed to generalise directly to the present adult sample, they suggest that a brief global trust measure may obscure domain-specific relationships. Accordingly, the absence of moderation should not be interpreted as evidence that trust is unimportant or that greater trust is necessarily beneficial. Future studies should compare direct, mediating, and moderating models using multidimensional, nutrition-specific measures of trust [31,32,37,43].

Acceptance of an AI-generated diet likewise did not significantly moderate the association between positive attitudes toward AI and e-HDL. This finding should not be interpreted as indicating that acceptance is unimportant in the use of AI-generated dietary recommendations. One possible explanation is that acceptance and e-HDL reflect different stages of engagement with nutrition information. In a systematic review of personalised dietary advice, Reinders et al. distinguished the generation and delivery of dietary advice from the subsequent stages of advice acceptance and adherence [59]. This framework suggests that acceptance is better conceptualised as a response to a specific recommendation than as a general competency in locating, understanding, appraising, and applying digital nutrition information. Acceptance may also depend on characteristics of the recommendation process itself. In an AI-based food recommender system, Vandeputte et al. found greater acceptance of food-substitution suggestions when users were actively involved in selecting the recommendation [28]. These findings suggest that acceptance may be more closely related to the characteristics and presentation of a specific recommendation than to the broader competencies represented by e-HDL. This interpretation remains tentative because the present study assessed stated willingness to accept an AI-generated diet rather than responses to actual AI-generated dietary recommendations.

From a practical perspective, attitudes toward AI may be relevant when considering how adults engage with digital nutrition information, although their contribution to e-HDL was modest. Dietitians, nutrition educators, and public health professionals should therefore avoid assuming that positive attitudes toward AI necessarily reflect adequate ability to critically evaluate AI-generated nutrition information or willingness to follow personalised AI-generated dietary advice. Technology-acceptance research further suggests that engagement with AI systems is influenced by technology-specific perceptions such as perceived usefulness and ease of use [33]. Accordingly, nutrition education and AI-supported nutrition initiatives may benefit from combining critical appraisal of digital nutrition information with opportunities to evaluate the usefulness, limitations, and appropriate role of AI tools. Clear communication regarding the limitations of AI-generated information and the continued role of professional evaluation may also be particularly important.

### Strengths and Limitations

This study addresses a timely and relatively underexplored question concerning the relationship between general attitudes towards AI and e-HDL. Examining positive and negative AI attitudes separately preserved the multidimensional structure of the construct, while the relatively large sample and use of previously validated instruments provided a sound basis for the analyses. Combining correlation analyses with PLS-SEM enabled assessment of both bivariate and multivariable structural relationships, and evaluation of the measurement model provided sample-specific evidence of reliability and construct validity. Mediation and moderation analyses further enabled theoretically proposed pathways to be tested, including relationships that were not supported by the data. Descriptive information on digital nutrition-information seeking, familiarity with AI applications, trust in AI-generated nutrition information, and willingness to accept an AI-generated diet also provided useful context for interpreting the principal findings.

Several limitations should be considered. The cross-sectional design precludes conclusions regarding temporal ordering or causality and is particularly restrictive for interpreting the proposed indirect pathways. Future longitudinal and experimental studies are needed to examine temporal and causal relationships and to validate the observed structural pathways. All data were self-reported and collected within a single survey, creating the possibility of recall error, social-desirability bias, and common method bias. Although the principal constructs were assessed using validated instruments, familiarity with AI, trust in AI-generated nutrition information, and willingness to accept an AI-generated diet were measured using brief self-report items that may not capture their multidimensional and context-dependent nature. These single-item measures may also be susceptible to measurement error and response bias. Residual confounding by unmeasured or incompletely measured factors, including general digital competence, broader health literacy, and previous AI experience, cannot be excluded. Body weight and height were also self-reported, and therefore the BMI measure may be subject to reporting bias, which is particularly relevant because BMI was examined as a potential mediator.

The use of convenience sampling from a single city (Kırıkkale) over a two-month period also limits the external validity of the findings and may introduce self-selection bias. The cultural context, educational characteristics, and local digital infrastructure may have influenced participants’ AI attitudes and e-HDL, limiting the generalizability of the findings to other regions and populations. In addition, the exclusion of dietitians and undergraduate nutrition and dietetics students may have influenced the distribution of AI attitudes and e-HDL, as participants with greater exposure to nutrition science were systematically excluded. Therefore, the findings should be interpreted cautiously when generalizing to broader and more diverse populations. Although the measurement properties were generally acceptable, some values were close to the recommended thresholds, including the Cronbach’s alpha for the e-HDLQ Understanding subscale (0.670) and the AVE for the Digital Healthy Diet Literacy subscale (0.418). Finally, because AI technologies and public engagement with them are evolving rapidly, the attitudes captured during data collection may change over time.

## 5. Conclusions

Positive attitudes towards AI were positively associated with e-HDL, whereas negative attitudes towards AI were inversely associated with e-HDL. Although these associations were statistically significant, the model explained only 12.2% of the variance in e-HDL, indicating that AI attitudes represent one of several factors associated with e-HDL rather than a dominant explanatory factor. Neither BMI nor the number of diagnosed diseases showed a significant indirect effect in the exploratory mediation analyses, and neither trust in AI-generated nutrition information nor willingness to accept an AI-generated diet significantly moderated the association between positive attitudes towards AI and e-HDL. Descriptively, a greater proportion of participants trusted AI-generated healthy nutrition information than were willing to accept an AI-generated nutrition/diet plan, which may reflect greater acceptance of AI as an information source than as a provider of personalised dietary recommendations. Longitudinal and intervention studies are needed to clarify temporal relationships and to determine whether these associations can be modified through targeted interventions.

## Figures and Tables

**Table 1:** General characteristics of participants (n = 610).

Variables	$\bar{X}$ ± SD
**Age (years)**	33.1 ± 11.4
**Number of diagnosed diseases**	1.4 ± 0.9
**BMI (kg/m^2^)**	25.3 ± 5.3
**GAAIS**	
Positive attitude	39.6 ± 10.2
Negative attitude	16.2 ± 4.4
**e-HDLQ**	
Finding e-healthy diet information	7.1 ± 2.8
Understanding e-healthy diet information	16.5 ± 2.3
Judging e-healthy diet information	7.2 ± 2.3
Applying e-healthy diet information	4.1 ± 2.0
Digital healthy diet literacy	10.2 ± 2.8
Total score	45.0 ± 6.8
	**n (%)**
**Gender**	
Female	310 (50.8)
Male	300 (49.2)
**Education level**	
Primary school	19 (3.2)
Middle school	35 (5.7)
High school	121 (19.8)
University	366 (60.0)
Master’s degree/Doctorate	69 (11.3)
**Marital status**	
Married	309 (50.7)
Single	301 (49.3)
**Income status**	
Income less than expenses	188 (30.8)
Income equal to expenses	270 (44.3)
Income more than expenses	152 (24.9)
**Diagnosed disease status**	
Yes	162 (26.6)
No	448 (73.4)
**BMI Classification**	
Underweight	42 (6.9)
Normal weight	271 (44.4)
Overweight	201 (33.0)
Class I obesity	74 (12.1)
Class II obesity	13 (2.1)
Class III obesity	9 (1.5)

Data are presented as mean ± standard deviation or n (%). BMI: body mass index; e-HDLQ: e-Healthy Diet Literacy Questionnaire; GAAIS: General Attitudes toward Artificial Intelligence Scale.

**Table 2:** Participants’ knowledge and attitudes regarding healthy nutrition on digital platforms and the use of AI in the field of nutrition and dietetics (n = 610).

	n (%)
**Importance given to the scientific nature of information on digital platforms**	
Give great importance	248 (40.7)
Give some importance	284 (46.6)
Give no importance	78 (12.7)
**Following dietitians’ digital platforms**	
Yes	338 (55.4)
No	272 (44.6)
**Effect of information obtained from digital platforms on healthy lifestyle habits**	
Very effective	123 (20.2)
Somewhat effective	350 (57.4)
Not very effective	110 (18.0)
Not effective at all	27 (4.4)
Topics of information obtained from digital sources *	
Effects of foods on health	320 (52.5)
Diet programs	197 (32.3)
Healthy recipes	306 (50.2)
Nutrition/diet recommendations for diseases (e.g., diabetes, heart disease, etc.)	166 (27.2)
Food supplements	231 (37.9)
Relationship between nutrition and body weight loss	239 (39.2)
Other	8 (1.3)
**Level of knowledge about AI applications in nutrition and dietetics**	
No knowledge at all	239 (39.2)
Very little knowledge	200 (32.8)
Undecided	91 (14.9)
Good level of knowledge	65 (10.7)
Very good level of knowledge	15 (2.4)
**Acceptance of implementing a diet prepared by AI**	
Yes	210 (34.4)
No	400 (65.6)
**Trust in the accuracy of healthy nutrition information obtained from AI**	
Trust	362 (59.3)
Do not trust	248 (40.7)
**Desire for the widespread use of AI-supported nutrition and dietetics applications**	
Yes	239 (39.2)
No	128 (21.0)
Undecided	243 (39.8)

* More than one option was marked. AI: artificial intelligence.

**Table 3:** Correlations between selected variables of the participants.

Variables	1	2	3	4	5	6	7	8	9	10
	r (*p*)	r (*p*)	r (*p*)	r (*p*)	r (*p*)	r (*p*)	r (*p*)	r (*p*)	r (*p*)	r (*p*)
**1. Age**	1	−0.034 (0.398)	0.118 (0.004) **	−0.011 (0.792)	0.025 (0.536)	−0.062 (0.125)	0.005 (0.899)	−0.119 (0.003) **	−0.135 (0.001) **	0.348 (<0.001) **
**2. e-HDLQ ** **Finding e-healthy diet information**		1	0.156 (<0.001) **	0.124 (0.002) **	0.450 (<0.001) **	0.273 (<0.001) **	0.744 (<0.001) **	−0.159 (<0.001) **	0.072 (0.077)	−0.028 (0.483)
**3. e-HDLQ ** **Understanding e-healthy diet information**			1	0.070 (0.084)	0.013 (0.749)	−0.026 (0.522)	0.425 (<0.001) **	0.036 (0.375)	0.020 (0.629)	0.030 (0.459)
**4. e-HDLQ ** **Judging e-healthy diet information**				1	0.036 (0.370)	0.100 (0.013) *	0.461 (<0.001) **	−0.151 (<0.001) **	0.303 (<0.001) **	0.046 (0.254)
**5. e-HDLQ ** **Applying e-healthy diet information**					1	0.134 (<0.001) **	0.547 (<0.001) **	−0.163 (<0.001) **	−0.016 (0.688)	−0.093 (0.021) *
**6. e-HDLQ ** **Digital healthy diet literacy**						1	0.582 (<0.001) **	−0.036 (0.379)	0.220 (<0.001) **	−0.062 (0.128)
**7. e-HDLQ ** **Total**							1	−0.165 (<0.001) **	0.222 (<0.001) **	−0.038 (0.347)
**8. GAAIS ** **Negative attitude**								1	−0.018 (0.661)	−0.149 (<0.001) **
**9. GAAIS ** **Positive attitude**									1	−0.049 (0.224)
**10. BMI**										1

BMI: body mass index; e-HDLQ: e-Healthy Diet Literacy Questionnaire; GAAIS: General Attitudes towards Artificial Intelligence Scale. Pearson correlation analysis. Statistically significant results are shown in bold. * *p* < 0.05, ** *p* < 0.001.

**Table 4:** Internal consistency reliability and convergent validity findings of the study variables.

Variables	Cronbach’s Alpha	Composite Reliability (rho_A)	Composite Reliability (CR)	Average Variance Extracted (AVE)
GAAIS negative attitude	0.815	0.724	0.846	0.534
GAAIS positive attitude	0.914	0.915	0.917	0.482
e-HDLQ Finding e-healthy diet information	0.789	0.790	0.877	0.704
e-HDLQ Understanding e-healthy diet information	0.670	0.722	0.794	0.494
e-HDLQ Judging e-healthy diet information	0.768	0.775	0.896	0.811
e-HDLQ Applying e-healthy diet information	0.689	0.701	0.865	0.762
e-HDLQ Digital healthy diet literacy	0.706	0.778	0.771	0.418
e-HDLQ total	0.729	0.760	0.825	0.543

e-HDLQ: e-Healthy Diet Literacy Questionnaire; GAAIS: General Attitudes towards Artificial Intelligence Scale; CR: composite reliability; AVE: average variance extracted. Note. The final measurement model included 17 GAAIS items, comprising 12 items for the Positive GAAIS and 5 items for the Negative GAAIS, following the removal of items 13, 14, and 16 due to low factor loadings.

**Table 5:** Discriminant validity results based on the HTMT ratios and Fornell–Larcker criterion.

	GAAIS Negative Attitude	GAAIS Positive Attitude	e-HDLQ Finding e-Healthy Diet Information	e-HDLQ Understanding e-Healthy Diet Information	e-HDLQ Judging e-Healthy Diet Information	e-HDLQ Applying e-Healthy Diet Information	e-HDLQ Digital Healthy Diet Literacy
**HTMT Ratios**							
**GAAIS negative attitude**							
**GAAIS positive attitude**	0.160						
**e-HDLQ ** **Finding e-healthy diet information**	0.224	0.144					
**e-HDLQ ** **Understanding e-healthy diet information**	0.107	0.123	0.213				
**e-HDLQ ** **Judging e-healthy diet information**	0.212	0.365	0.163	0.096			
**e-HDLQ ** **Applying e-healthy diet information**	0.247	0.160	0.610	0.143	0.119		
**e-HDLQ ** **Digital healthy diet literacy**	0.127	0.275	0.362	0.171	0.163	0.285	
**Fornell–Larcker Criterion**							
**GAAIS negative attitude**	0.730						
**GAAIS positive attitude**	−0.083	0.695					
**e-HDLQ ** **Finding e-healthy diet information**	−0.220	0.144	0.839				
**e-HDLQ ** **Understanding e-healthy diet information**	−0.027	0.069	0.166	0.703			
**e-HDLQ ** **Judging e-healthy diet information**	−0.168	0.311	0.126	0.064	0.901		
**e-HDLQ ** **Applying e-healthy diet information**	−0.224	0.073	0.453	0.041	0.040	0.873	
**e-HDLQ ** **Digital healthy diet literacy**	−0.077	0.245	0.307	0.015	0.109	0.180	0.647

HTMT: heterotrait–monotrait ratio; e-HDLQ: e-Healthy Diet Literacy Questionnaire; GAAIS: General Attitudes toward Artificial Intelligence Scale.

**Table 6:** Explanatory power, predictive relevance, and effect size results for the structural model.

Variables	R^2^	Adjusted R^2^	Q^2^	*f* ^2^
e-HDLQ	0.122	0.120	0.109	
**Relationship between variables**				
GAAIS negative attitude → e-HDLQ				0.061
GAAIS positive attitude → e-HDLQ				0.067
e-HDLQ → Finding e-healthy diet information				2.468
e-HDLQ → Understanding e-healthy diet information				0.083
e-HDLQ → Judging e-healthy diet information				0.106
e-HDLQ → Applying e-healthy diet information				0.697
e-HDLQ → Digital healthy diet literacy				0.680

e-HDLQ: e-Healthy Diet Literacy Questionnaire; GAAIS: General Attitudes toward Artificial Intelligence Scale; R^2^: coefficient of determination (explained variance); Q^2^: Stone–Geisser predictive relevance coefficient; *f*^2^: effect size.

**Table 7:** Structural model path coefficients and hypothesis test results.

Path	Original Sample (O)	Sample Mean (M)	SD	t	*p*
GAAIS negative attitude → e-HDLQ	−0.232	−0.235	0.037	6.229	<0.001 ***
GAAIS positive attitude → e-HDLQ	0.243	0.257	0.032	7.692	<0.001 ***

e-HDLQ: e-Healthy Diet Literacy Questionnaire; GAAIS: General Attitudes towards Artificial Intelligence Scale; SD: standard deviation; t: t-statistic; *p*: *p*-value. Statistically significant results are shown in bold. *** *p* < 0.001.

**Table 8:** PROCESS Macro analysis results for the indirect effects through BMI and number of diagnosed diseases.

Path 1: Mediation of BMI	B	SH	t	*p*	%95 Cl (LL–UL)
GAAIS positive attitude → BMI (a path)	−0.026	0.021	−1.217	0.224	−0.067–0.016
BMI → e-HDLQ (b path)	−0.035	0.051	−0.689	0.491	−0.136–0.065
GAAIS positive attitude → e-HDLQ (c′ direct effect)	0.148	0.027	5.577	<0.001	0.096–0.200
GAAIS positive attitude → e-HDLQ (c total effect)	0.149	0.026	5.620	<0.001	0.097–0.201
**Indirect effect (X → BMI → Y)**	0.001	0.002	—	—	−0.003–0.006
**Model Summary**	**R**		**R^2^**	**F**	***p***
GAAIS positive attitude → BMI	0.049		0.002	1.482	0.224
GAAIS positive attitude & BMI → e-HDLQ	0.224		0.050	16.018	<0.001
Total effect model	0.222		0.049	31.588	<0.001
**Path 2: Mediation of Number of Diagnosed Diseases Effect**	**B**	**SH**	**t**	***p***	**%95 Cl (LL–UL)**
GAAIS positive attitude → Number of diagnosed diseases (a path)	0.001	0.007	0.173	0.863	−0.012–0.014
Number of diagnosed diseases → e-HDLQ (b path)	−0.411	0.540	−0.761	0.448	−1.477–0.656
GAAIS positive attitude → e-HDLQ (c′ direct effect)	0.142	0.044	3.209	0.002	0.055–0.229
GAAIS positive attitude → e-HDLQ (c total effect)	0.141	0.044	3.203	0.002	0.054–0.229
**Indirect effect (X → Number of Diagnosed Diseases → Y)**	−0.001	0.005	—	—	−0.015–0.005
**Model Summary**	**R**		**R^2^**	**F**	***p***
GAAIS positive attitude → Number of diagnosed diseases	0.014		0.000	0.030	0.863
GAAIS positive attitude & Number of diagnosed diseases → e-HDLQ	0.252		0.064	5.405	0.005
Total effect model	0.246		0.060	10.259	0.002

BMI: body mass index; e-HDLQ: e-Healthy Diet Literacy Questionnaire; GAAIS: General Attitudes towards Artificial Intelligence Scale; B: unstandardized regression coefficient; SE: standard error; t: t-statistic; CI: confidence interval; LL: lower limit; UL: upper limit; R: multiple correlation coefficient; R^2^: coefficient of determination; F: F-statistic. For indirect effects, statistical significance was determined based on the 95% bootstrap confidence intervals (CI) with 5000 resamples. An indirect effect is considered statistically significant if the confidence interval does not include zero.

**Table 9:** PROCESS Macro analysis results for the moderating effects of AI-related variables on the relationship between positive attitudes towards AI and e-HDLQ.

Moderator 1: Acceptance of an AI-Generated Nutrition/Diet Plan								
Predictor				**B**	**SE**	**t**	***p***	**95% CI (LL–UL)**
Constant				40.117	1.323	30.328	<0.001	37.519–42.715
GAAIS positive attitude (X)				0.116	0.034	3.413	0.001	0.049–0.183
Acceptance of an AI-generated nutrition/diet plan (W1)				−1.750	2.447	−0.715	0.475	−6.555–3.056
GAAIS positive attitude × acceptance of an AI-generated nutrition/diet plan (X × W1)				0.062	0.057	1.078	0.281	−0.051–0.175
**Model Summary**				Change in R^2^ Due to Interaction				
**R**	**R^2^**	**F**	***p***	**Interaction**		**ΔR^2^**	**F**	***p***
0.233	0.054	11.574	<0.001	X × W1		0.002	1.162	0.281
**Moderator 2: Trust in the Accuracy of AI-Generated Healthy Nutrition Information**								
Predictor				**B**	**SE**	**t**	***p***	**95% CI (LL–UL)**
Constant				40.579	1.786	22.725	<0.001	37.072–44.086
GAAIS positive attitude (X)				0.105	0.047	2.220	0.027	0.012–0.198
Trust in the accuracy of AI-generated healthy nutrition information (W3)				−1.958	2.287	−0.856	0.392	−6.449–2.533
GAAIS positive attitude × trust in the accuracy of AI-generated healthy nutrition information (X × W3)				0.059	0.058	1.023	0.307	−0.055–0.173
**Model Summary**				Change in R^2^ Due to Interaction				
**R**	**R^2^**	**F**	***p***	**Interaction**		**ΔR^2^**	**F**	***p***
0.227	0.051	10.967	<0.001	X × W3		0.002	1.047	0.307

GAAIS: General Attitudes towards Artificial Intelligence Scale; B: unstandardized regression coefficient; SE: standard error; t: t-statistic; CI: confidence interval; LL: lower limit; UL: upper limit; R: multiple correlation coefficient; R^2^: coefficient of determination; ΔR^2^: change in R^2^ when the interaction term is added to the model; F: F-statistic.

## Data Availability

The data presented in this study are available on request from the corresponding author due to privacy.

## Supplementary Materials

The following supporting information can be downloaded at: https://www.mdpi.com/article/10.3390/nu18172931/s1, Table S1: Factor loadings and variance inflation factor (VIF) values of the scales used in the study.

## Abbreviations

The following abbreviations are used in this manuscript:

e-HDL	e-healthy diet literacy
AI	artificial intelligence
e-HDLQ	e-Healthy Diet Literacy Questionnaire
PLS-SEM	Partial Least Squares Structural Equation Modeling
BMI	body mass index
WHO	World Health Organization
GAAIS	General Attitudes toward Artificial Intelligence Scale
VIF	variance inflation factor

## References

[1] Spronk I., Kullen C., Burdon C., O’Connor H. (2014). Relationship between Nutrition Knowledge and Dietary Intake. Br. J. Nutr..

[2] Wardle J., Parmenter K., Waller J. (2000). Nutrition Knowledge and Food Intake. Appetite.

[3] Franklin J., Holman C., Tam R., Gifford J., Prvan T., Stuart-Smith W., Denyer G., Markovic T., O’Connor H. (2020). Validation of the E-NutLit, an Electronic Tool to Assess Nutrition Literacy. J. Nutr. Educ. Behav..

[4] Sørensen K., Van Den Broucke S., Fullam J., Doyle G., Pelikan J., Slonska Z., Brand H. (2012). Health Literacy and Public Health: A Systematic Review and Integration of Definitions and Models. BMC Public Health.

[5] Van Duong T., Chiu C.-H., Lin C.-Y., Chen Y.-C., Wong T.-C., Chang P.W.S., Yang S.-H. (2021). E-Healthy Diet Literacy Scale and Its Relationship with Behaviors and Health Outcomes in Taiwan. Health Promot. Int..

[6] Denniss E., Lindberg R., McNaughton S.A. (2023). Quality and Accuracy of Online Nutrition-Related Information: A Systematic Review of Content Analysis Studies. Public Health Nutr..

[7] Shaik T., Tao X., Higgins N., Li L., Gururajan R., Zhou X., Acharya U.R. (2023). Remote Patient Monitoring Using Artificial Intelligence: Current State, Applications, and Challenges. Wiley Interdiscip. Rev. Data Min. Knowl. Discov..

[8] Gansser O.A., Reich C.S. (2021). A New Acceptance Model for Artificial Intelligence with Extensions to UTAUT2: An Empirical Study in Three Segments of Application. Technol. Soc..

[9] Luan H., Geczy P., Lai H., Gobert J., Yang S.J.H., Ogata H., Baltes J., Guerra R., Li P., Tsai C.C. (2020). Challenges and Future Directions of Big Data and Artificial Intelligence in Education. Front. Psychol..

[10] Yang Z., Khatibi E., Nagesh N., Abbasian M., Azimi I., Jain R., Rahmani A.M. (2024). ChatDiet: Empowering Personalized Nutrition-Oriented Food Recommender Chatbots through an LLM-Augmented Framework. Smart Health.

[11] Neudert L.M., Knuutila A., Howard P.N. (2020). Global Attitudes Towards AI, Machine Learning & Automated Decision Making: Implications for Involving Artificial Intelligence in Public Service and Good Governance.

[12] Davis F.D. (1989). Perceived Usefulness, Perceived Ease of Use, and User Acceptance of Information Technology. MIS Q..

[13] Venkatesh V., Morris M.G., Davis G.B., Davis F.D. (2003). User Acceptance of Information Technology: Toward a Unified View. MIS Q..

[14] Schepman A., Rodway P. (2020). Initial Validation of the General Attitudes towards Artificial Intelligence Scale. Comput. Hum. Behav. Rep..

[15] Schepman A., Rodway P. (2023). The General Attitudes towards Artificial Intelligence Scale (GAAIS): Confirmatory Validation and Associations with Personality, Corporate Distrust, and General Trust. Int. J. Hum. Comput. Interact..

[16] Güler Ü., Mengї Çelїk Ö., Başpınar E.N., Değїrmencїoğlu E., Eser B., Serenlїoğlu D., Kılıç G., Gençoğlu İ., Kaya M., Sarıteke Y. (2026). Associations between Digital Healthy Diet Literacy, Artificial Intelligence Attitudes, Mediterranean Diet Adherence, and Health-Related Quality of Life in Adults. BMC Public Health.

[17] Adiyan N.N., Lule N.O. (2026). Attitudes toward Artificial Intelligence Tools in University Students: Associations with Body Appreciation and e-Healthy Diet Literacy—Implications for Digital Health Education. BMC Med. Educ..

[18] World Medical Association WMA Declaration of Helsinki-Ethical Principles for Medical Research Involving Human Participants. Cited: 4 August 2025. https://www.wma.net/policies-post/wma-declaration-of-helsinki/.

[19] Body Mass Index (BMI). https://www.who.int/data/gho/data/themes/topics/topic-details/GHO/body-mass-index.

[20] Onbaşı Ö., Türker P.F. (2023). E-Sağlıklı Beslenme Okuryazarlığı Ölçeğinin Geçerlik ve Güvenirliğinin İncelenmesi. J. Nutr. Diet..

[21] Kaya F., Aydin F., Schepman A., Rodway P., Yetişensoy O., Demir Kaya M. (2024). The Roles of Personality Traits, AI Anxiety, and Demographic Factors in Attitudes toward Artificial Intelligence. Int. J. Hum. Comput. Interact..

[22] Chan Y. (2003). Biostatistics 101: Data Presentation. Singap. Med. J..

[23] Shao A.T. (2002). Marketing Research: An Aid to Decision Making.

[24] Hair J.F., Risher J.J., Sarstedt M., Ringle C.M. (2019). When to Use and How to Report the Results of PLS-SEM. Eur. Bus. Rev..

[25] Hair J.F., Ringle C.M., Sarstedt M. (2011). PLS-SEM: Indeed a Silver Bullet. J. Mark. Theory Pract..

[26] Doğan İ. (2015). Farklı Veri Yapısı ve Örneklem Büyüklüklerinde Yapısal Eşitlik Modellerinin Geçerliği ve Güvenirliğinin Değerlendirilmesi.

[27] Cohen J. (1988). Statistical Power Analysis for the Behavioral Sciences.

[28] Vandeputte J., Herold P., Kuslii M., Viappiani P., Muller L., Martin C., Davidenko O., Delaere F., Manfredotti C., Cornuéjols A. (2023). Principles and Validations of an Artificial Intelligence-Based Recommender System Suggesting Acceptable Food Changes. J. Nutr..

[29] Reinders M.J., Bouwman E.P., Van Den Puttelaar J., Verain M.C.D. (2020). Consumer Acceptance of Personalised Nutrition: The Role of Ambivalent Feelings and Eating Context. PLoS ONE.

[30] Delibasi S.I., Hayyar S., Bektas H. (2026). Digital Readiness in Nursing Education: EHealth Literacy, AI Attitudes, and Associated Factors among Undergraduate Students. Nurse Educ. Today.

[31] Shin D. (2021). The Effects of Explainability and Causability on Perception, Trust, and Acceptance: Implications for Explainable AI. Int. J. Hum. Comput. Stud..

[32] Glikson E., Woolley A.W. (2020). Human Trust in Artificial Intelligence: Review of Empirical Research. Acad. Manag. Ann..

[33] Ibrahim F., Münscher J.C., Daseking M., Telle N.T. (2024). The Technology Acceptance Model and Adopter Type Analysis in the Context of Artificial Intelligence. Front. Artif. Intell..

[34] Norman C.D., Skinner H.A. (2006). EHealth Literacy: Essential Skills for Consumer Health in a Networked World. J. Med. Internet Res..

[35] Boztepe H., Akdeniz Kudubeş A., Semerci Şahin R., Durmuş Sarıkahya S., Çınar Özbay S. (2025). Examining the Relationship between Nursing Students’ Readiness, Literacy and Attitudes toward Medical Artificial Intelligence. Nurse Educ. Pract..

[36] Paige S.R., Stellefson M., Krieger J.L., Anderson-Lewis C., Cheong J.W., Stopka C. (2018). Proposing a Transactional Model of EHealth Literacy: Concept Analysis. J. Med. Internet Res..

[37] Reinhardt A., Matthes J., Hodzic S., Kaňková J., Bojic L., Maindal H.T., Paraschiv C., Ryom K. (2025). Who Trusts AI for Health Information? A Cross-National Survey on Trust Determinants in Four European Countries. Health Commun..

[38] Ruani M.A., Reiss M.J., Kalea A.Z. (2023). Diet-Nutrition Information Seeking, Source Trustworthiness, and Eating Behavior Changes: An International Web-Based Survey. Nutrients.

[39] Rosenbacke R., Melhus Å., McKee M., Stuckler D. (2024). How Explainable Artificial Intelligence Can Increase or Decrease Clinicians’ Trust in AI Applications in Health Care: Systematic Review. JMIR AI.

[40] Biagini G. (2025). Towards an AI-Literate Future: A Systematic Literature Review Exploring Education, Ethics, and Applications. Int. J. Artif. Intell. Educ..

[41] Zhang S., Ganapathy Prasad P., Schroeder N.L. (2025). Learning About AI: A Systematic Review of Reviews on AI Literacy. J. Educ. Comput. Res..

[42] Yang Y., Zhang Y., Sun D., He W., Wei Y. (2025). Navigating the Landscape of AI Literacy Education: Insights from a Decade of Research (2014–2024). Humanit. Soc. Sci. Commun..

[43] Everett J.A.C., Claessens S., Knöchel T.-D., Reinecke M.G. (2026). Principles for Understanding Trust in Artificial Intelligence. Nat. Rev. Psychol..

[44] Catapan S.d.C., Sazon H., Zheng S., Gallegos-Rejas V., Mendis R., Santiago P.H.R., Kelly J.T. (2025). A Systematic Review of Consumers’ and Healthcare Professionals’ Trust in Digital Healthcare. npj Digit. Med..

[45] Wendel S., Dellaert B.G., Ronteltap A., van Trijp H.C. (2013). Consumers’ Intention to Use Health Recommendation Systems to Receive Personalized Nutrition Advice. BMC Health Serv. Res..

[46] Zhu X., Stroud A.M., Minteer S.A., Yoo D.W., Ridgeway J.L., Mooghali M., Miller J.E., Barry B.A. (2026). Key Information Influencing Patient Decision-Making About AI in Health Care: Survey Experiment Study. J. Med. Internet Res..

[47] Yıldız Kopuz T.N., Haydaroğlu M., Uçar Baş K. (2026). Development and Validation of the Dietitians’ Perceived Trust in Artificial Intelligence Scale: A Multidimensional Measure for Professional Practice. Nutr. Diet..

[48] Isaksen A.A., Schaarup J.R., Bjerg L., Hulman A. (2025). Changes in Public Perception of Artificial Intelligence in Healthcare after Exposure to ChatGPT. npj Digit. Med..

[49] Haman M., Školník M., Lošťák M. (2024). AI Dietician: Unveiling the Accuracy of ChatGPT’s Nutritional Estimations. Nutrition.

[50] Liao L.L., Chang L.C., Lai I.J. (2024). Assessing the Quality of ChatGPT’s Dietary Advice for College Students from Dietitians’ Perspectives. Nutrients.

[51] Cicero L., Russo A., Di Stefano G., Zammitti A. (2025). The General Attitudes towards Artificial Intelligence Scale (GAAIS): Validation and Psychometric Properties Analysis in the Italian Context. BMC Psychol..

[52] Rózsa S., Bandi S., Hartung I., Török I.A., Varga J., Somlai E.H., Herold R., Kállai J. (2025). General Attitudes towards Artificial Intelligence Scale (GAAIS): Hungarian Adaptation and Links to Personality Traits. Front. Psychol..

[53] Hua Z., Yuqing S., Qianwen L., Hong C. (2025). Factors Influencing EHealth Literacy Worldwide: Systematic Review and Meta-Analysis. J. Med. Internet Res..

[54] Lee A.T., Ramasamy R.K., Subbarao A. (2025). Understanding Psychosocial Barriers to Healthcare Technology Adoption: A Review of TAM Technology Acceptance Model and Unified Theory of Acceptance and Use of Technology and UTAUT Frameworks. Healthcare.

[55] Jung F.U., Luppa M., Reusche M., Wirkner K., Eberl M., Dietz Y., Engel C., Riedel-Heller S.G. (2026). Understanding Digital Health Literacy in Later Life: The Role of Sociodemographic and Health-Related Factors—A Cross-Sectional Study. BMC Geriatr..

[56] Madrigal L., Escoffery C. (2019). Electronic Health Behaviors Among US Adults With Chronic Disease: Cross-Sectional Survey. J. Med. Internet Res..

[57] Wang Z., Fan Y., Lv H., Deng S., Xie H., Zhang L., Luo A., Wang F. (2022). The Gap Between Self-Rated Health Information Literacy and Internet Health Information-Seeking Ability for Patients With Chronic Diseases in Rural Communities: Cross-Sectional Study. J. Med. Internet Res..

[58] Cicin F.N., Çetin Gürkan G. (2026). How Physicians Embrace AI: Insights from Technology Acceptance and Trust Theories. Front. Digit. Health.

[59] Reinders M.J., Starke A.D., Fischer A.R.H., Verain M.C.D., Doets E.L., Van Loo E.J. (2023). Determinants of Consumer Acceptance and Use of Personalized Dietary Advice: A Systematic Review. Trends Food Sci. Technol..

