# Supplementary material for: Positive and Negative Attitudes Toward Artificial Intelligence and E-Healthy Diet Literacy Among Adults: A Partial Least Squares Structural Equation Modeling Study
Source: Nutrients. 2026 Sep 7;18(17):2931. doi: 10.3390/nu18172931 (PMC13567288; doi:10.3390/nu18172931)
Supplement: Supplementary file 1 [file nutrients-18-02931-s001.zip › nutrients-4498305-supplementary.pdf]

Supplementary Table S1. Factor loadings and variance inflation factor (VIF) values of the scales used in the study.

	VIF	Factor Loading						
		e-HDLQ Digital Healthy Diet Literacy	GAAIS Negative attitude	GAAIS Positive attitude	e-HDLQ Finding e-Healthy Diet Information	e-HDLQ Understandin g e-Healthy Diet Information	e-HDLQ Judging e-Healthy Diet Information	e-HDLQ Applying e-Healthy Diet Information
**e-HDL12**	1.379	0.597						
**e-HDL13**	1.607	0.820						
**e-HDL14**	1.496	0.728						
**e-HDL15**	1.543	0.784						
**GAAIS15**	1.404		0.423					
**GAAIS17**	1.978		0.757					
**GAAIS18**	1.689		0.796					
**GAAIS19**	1.517		0.785					
**GAAIS20**	1.907		0.816					
**GAAIS1**	1.863			0.644				
**GAAIS2**	2.694			0.720				
**GAAIS3**	2.853			0.635				
**GAAIS4**	2.105			0.681				
**GAAIS5**	2.072			0.685				
**GAAIS6**	1.856			0.683				
**GAAIS7**	2.527			0.683				
**GAAIS8**	2.113			0.801				
**GAAIS9**	1.865			0.753				
**GAAIS10**	1.848			0.717				
**GAAIS11**	1.896			0.735				
**GAAIS12**	1.648			0.569				
**e-HDL1**	1.509				0.811			
**e-HDL2**	2.004				0.853			
**e-HDL3**	1.900				0.852			

**e-HDL4**	1.263	0.807		
**e-HDL5**	1.305	0.707		
**e-HDL6**	1.374	0.698		
**e-HDL7**	1.290	0.580		
**e-HDL10**	1.382		0.851	
**e-HDL11**	1.382		0.894	
**e-HDL8**	1.884			0.913
**e-HDL9**	1.852			0.889

VIF: Variance inflation factor; e-HDLQ: e-Healthy Diet Literacy Questionnaire; GAAIS: General Attitudes toward Artificial Intelligence Scale.
